# Co-circulation of different *A. phagocytophilum* variants within cattle herds and possible reservoir role for cattle

**DOI:** 10.1186/s13071-018-2661-7

**Published:** 2018-03-09

**Authors:** Anne-Claire Lagrée, Clotilde Rouxel, Maëllys Kevin, Thibaud Dugat, Guillaume Girault, Benoît Durand, Martin Pfeffer, Cornelia Silaghi, Marion Nieder, Henri-Jean Boulouis, Nadia Haddad

**Affiliations:** 1 UMR BIPAR, Ecole Nationale Vétérinaire d’Alfort, Anses, INRA, Université Paris-Est, Maisons-Alfort, France; 2 Unité Zoonoses Bactériennes, Anses, Université Paris-Est, Maisons-Alfort, France; 3 Unité d’Epidémiologie, Anses, Université Paris-Est, Maisons-Alfort, France; 4 Institute of Animal Hygiene and Veterinary Public Health, Faculty of Veterinary Medicine, University of Leipzig, Leipzig, Germany; 5 Institute of Infectology, Friedrich-Loeffler-Institut, Federal Research Institute for Animal Health, Greifswald-Insel Riems, Germany

**Keywords:** *Anaplasma phagocytophilum*, Tick-borne disease, Bovine, France, Germany, MLST, Reservoir

## Abstract

**Background:**

*Anaplasma phagocytophilum* is a zoonotic tick-borne intracellular alpha-proteobacterium causing tick-borne fever, which leads to significant economic losses in domestic ruminants in Europe. Its epidemiological cycles are complex and reservoir host species of bovine strains have not yet been identified. Given that little genetic information is available on strains circulating within a defined bovine environment, our objective was to assess the genetic diversity of *A. phagocytophilum* obtained from the same farms over time.

**Methods:**

Blood samplings were performed several times in two European herds. In the French herd, 169 EDTA-blood samples were obtained from 115 cows (32 were sampled two to four times). In the German herd, 20 cows were sampled six times (120 EDTA-blood samples). The presence of *A. phagocytophilum* DNA was assessed using a qPCR targeting *msp2*. The positive DNA samples underwent MLST at nine genetic markers (*typA*, *ctrA*, *msp4*, *pleD*, *recG*, *polA*, *groEL*, *gyrA*, and *ankA*). For each locus, sequences were aligned with available bacterial sequences derived from cattle, horse, dog, and roe deer hosts, and concatenated neighbor joining trees were constructed using three to six loci.

**Results:**

Around 20% (57/289) of samples were positive. Forty positive samples from 23 French and six German cows (11 of them being positive at two time points) were sequenced. Six loci (*typA*, *ctrA*, *msp4*, *pleD*, *recG*, and *polA*) allowed to build concatenated phylogenetic trees, which led to two distinct groups of bovine variants in the French herd (hereafter called A and B), whereas only group A was detected in the German herd. In 42% of French samples, double chromatogram peaks were encountered in up to four loci. Eleven cows were found infected three weeks to 17 months after first sampling and harboured a new variant belonging to one or the other group.

**Conclusions:**

Our results demonstrate the occurrence of two major bovine strain groups and the simultaneous infection of single cows by more than one *A. phagocytophilum* strain. This challenges the role of cattle as reservoirs for *A. phagocytophilum.* This role may be facilitated via long-term bacterial persistence in individual cows and active circulation at the herd scale.

**Electronic supplementary material:**

The online version of this article (10.1186/s13071-018-2661-7) contains supplementary material, which is available to authorized users.

## Background

*Anaplasma phagocytophilum* is a zoonotic intracellular alpha-proteobacterium transmitted by ticks belonging to the *Ixodes persulcatus* complex. In Europe, the exophilic hard tick *Ixodes ricinus* is the main vector of *A. phagocytophilum* [1]. This bacterium infects a wide range of vertebrate hosts, including wild and domestic ruminants, dogs, horses, rodents and humans [2]. For decades it has been known that *A. phagocytophilum* causes tick-borne fever (TBF) in domestic ruminants in Europe [3, 4].

TBF outbreaks usually occur following the first time cattle graze in an endemic area [5]. The main clinical signs are hyperthermia, anorexia, mild to moderate depression, a sudden drop in milk yield, and abortions in pregnant females. Respiratory signs such as coughing, polypnea, nasal discharge, and abnormal lung sounds have also been described [6]. For European farms, this infection leads to significant economic losses [7], whereas TBF has never been described in the USA to date [8].

*Anaplasma phagocytophilum* epidemiological cycles are complex and differ greatly between the USA and Europe [8]. Several genetic lineages are described and some are thought to have specific host tropism [2]. Transovarial transmission has not yet been described in the vector tick under natural conditions [2]. Therefore, exploiting mammalian hosts as reservoirs is essential for *A. phagocytophilum* survival [8]. Several wild ruminant and rodent species are suspected to be involved [8].

In Europe, wild ruminants, especially red deer, are currently thought to carry strains infecting cattle [8–13]. Moreover, experimental infections in sheep - which are among the most frequently infected domestic species in Europe [14] - demonstrated long-term bacterial persistence in sheep peripheral blood [15], suggesting that they could also be reservoirs of sheep-infecting strains. However, proof that cattle play a role as reservoirs for cow-infecting strains has not yet been obtained.

The development of many novel molecular typing methods in recent years offers an unparalleled opportunity to generate new information about the complex epidemiology of *A. phagocytophilum* and its genetic diversity [8]. Among these techniques, multi-locus sequence typing (MLST), based on PCR amplification and sequencing of several housekeeping genes, is widely recognized as a reliable phylogenetic method for bacterial molecular characterization, specifically *A. phagocytophilum*, for which few other appropriate multi-loci typing methods are currently available [8, 9].

The objectives of this study were to explore the molecular epidemiology of *A. phagocytophilum* strains from cattle by longitudinally assessing their circulation, persistence, and genetic diversity in individual cattle and within herds from both France and Germany.

## Methods

### Animal sampling

#### French samples

In 2008, early clinical signs of disease, including fever, paresis, weight loss and abortions, and leading some animals to death, were observed in cattle (*Bos taurus*) in a farm near Gien, Sologne, France. Initial testing for intoxication returned negative results. When tick-borne fever was suggested as a possible cause, animals were treated with oxytetracycline without any further diagnostic investigation, and they recovered.

Since then, TBF has been continually clinically suspected on the farm, but clinical consequences have been less severe as animals were quickly treated. In October 2014, in March, May and October 2016, and in May 2017, blood sampling was preferentially performed on young cattle, and on cattle that were positive during previous samplings. In total, 169 EDTA-blood samples were obtained from 115 cows (Table 1). Thirty-two animals were sampled several times (between two to four times) during the study.

**Table 1:** Detection of *A. phagocytophilum* DNA by real-time PCR in cattle from Sologne, France

Date of sampling (year/month/day)	No. of cows sampled	No. of positive individuals (%)
2014/10/09	32	7 (21.9)
2016/03/03	34	6 (17.6)
2016/05/17	40	9 (22.5)
2016/10/26	34	3 (8.8)
2017/05/16	29	6 (20.7)
Total no. of samples	169	31 (18.3)

#### German samples

In 2009 and 2010, several cows from a farm in Meschede, in North-Rhine Westphalia, presented with high fever, decreased milk yield, lower limb edema, and anorexia, one to two weeks after having been brought to pasture. After initially suspecting a pasture infection, *A. phagocytophilum* was finally detected in the herd by PCR and blood smears [16]. In 2011, cows and heifers of the herd were sampled six times, for a period of up to five months, from the point at which they first developed fever [17]. In total, 120 EDTA-blood samples were used for this study (Table 2) [17].

**Table 2:** Detection of *A. phagocytophilum* DNA by real-time PCR in cattle from Meschede, Germany. The same 20 animals (including 19 heifers) were sampled over time

Date of sampling (year/month/day)	No. of cows sampled	No. of positive individuals (%)
2011/05/17–22	20	6 (30.0)
2011/05/30–2011/06/04	20	7 (35.0)
2011/06/14–22	20	7 (35.0)
2011/07/09	20	2 (10.0)
2011/09/07	20	3 (15.0)
2011/10/03	20	1 (5.0)
Total no. of samples	120	26 (21.7)

### DNA extraction and qPCR

DNA from EDTA-blood samples from French cattle was extracted using the NucleoSpin Blood QuickPure kit (Macherey-Nagel, Hoerdt, France), and DNA from German EDTA-blood samples with the Qiagen DNA Minikit (Qiagen, Hilden, Germany), both according to the manufacturer’s instructions. DNA extracts were stored at -20 °C prior to testing.

The presence of specific *A. phagocytophilum* DNA was assessed with a qPCR targeting a 77 bp fragment of the major surface protein 2 (*msp2*) gene, according to the protocol of Courtney et al. [18]. Positive (DNA from an *A. phagocytophilum* (Webster strain)-infected tick cell line IRE-CTVM20) and negative (molecular biology grade water) controls were included in each PCR run.

### Genotyping

In order to type the *A. phagocytophilum*-positive samples, the nine loci selected in a recently published *A. phagocytophilum* MLST study were used: *typA*, *ctrA* (APH 1099–1100), *msp4*, *pleD*, *recG*, *polA*, *groEL*, *gyrA*, and *ankA* [9]. PCRs for the nine loci were performed according to Chastagner et al. [9]. A nested PCR was then performed for all loci except *pleD*, using 5 μl of the initial PCR product. Amplicons were separated by electrophoresis on 1.5% agarose gels and stained with ethidium bromide for imaging. If the initial amplification yielded sufficient DNA of the correct size, this PCR product was used for sequencing. Otherwise, the shorter fragment obtained with internal primers was used. The bands were excised from the gel and purified using NucleoSpin Gel and PCR Clean-up kit (Macherey-Nagel), following the manufacturer’s instructions. Nucleotide sequences were obtained from PCR products by Sanger sequencing in both directions (Eurofins Genomics, Ivry-sur-Seine, France). Sequencing results were manually edited in Bioedit software version 7.2.5 (Ibis Biosciences, Carlsbad, USA). IUPAC codes were used to indicate ambiguous loci.

### Construction of phylogenetic trees

Nucleotide sequences were aligned using the MEGA7 software (Molecular Evolutionary Genetics Analysis version 7.0.18 [19]), with the ClustalW program. In order to build phylogenetic trees, the sequences of each locus obtained in this study were aligned with *A. phagocytophilum* sequences from cow, horse, dog, and roe deer hosts from Chastagner et al. [9] (GenBank: KJ832158–KJ833031) (Additional file 1: Table S1). Moreover, sequences of American human, dog, rodent, and tick *A. phagocytophilum* strains (HZ, Webster, HGE1, Dog2, JM and CRT), and a Norwegian sheep (*Ovis aries*) strain (Norway_variant2), were added to the alignments [9].

Phylogenetic trees were built after concatenation of three to six loci. Concatenated sequences were imported and neighbor joining trees (NJ trees) were constructed from the alignment using BioNumerics version 7.6.1 software (Applied Maths, Sint-Martens-Latem, Belgium). A profile is defined here as *A. phagocytophilum* sequences obtained from chromatograms for three to six loci. A phylogenetic tree based on the concatenation of six markers was built for samples with complete profiles, i.e. complete sequences without ambiguities of all six genetic markers, with the addition of concatenated sequences from 40 cows and two horses obtained by Chastagner et al. [9] and HZ, Webster, Dog2, JM, and Norway_variant2 strains.

Due to missing data or ambiguous sites for some loci, other trees based on the concatenation of five loci (*typA*, *ctrA*, *pleD*, *recG* and *polA*), four loci (*typA*, *ctrA*, *msp4* and *pleD*; *typA*, *ctrA*, msp4 and polA; *typA*, *ctrA*, *msp4* and *recG*), and three loci (*typA*, *ctrA* and *msp4*) were built. Other host species samples (six more horses, three dogs, five roe deer, HGE1, and CRT strains) from Chastagner et al. [9], which had generated sequences for only three or four loci, were added to the corresponding concatenated trees.

## Results

### Detection of *A. phagocytophilum* DNA

#### French samples

Altogether, 31/169 (18.3%) blood samples from 23 cattle (20/23 were heifers) were positive for specific *A. phagocytophilum* DNA with *msp2*-real-time PCR. The percentage of infected samples varied from 8.8% (October 2016) to 22.5% (May 2016) (Table 1). Of the 32 cows sampled at least twice during the experiment, eight yielded positive results at two successive sampling dates: four of them were positive two months apart between March and May 2016, three were positive after seven months between October 2016 and May 2017, and a bull was still found to be infected after 17 months (Table 3).

**Table 3:** French cattle found to be infected twice during the sampling period

Bovine ID	Sampling date (year/month/day)					Time span between the two positive samples (months)
	2014/10/09	2016/03/03	2016/05/17	2016/10/26	2017/05/16	
BV2802	Pos	Pos	Neg	na	na	17
BV0001	na	Pos	Pos	Neg	Neg	2.5
BV0006	na	Pos	Pos	Neg	Neg	2.5
BV0016	na	Pos	Pos	Neg	Neg	2.5
BV9949	na	Pos	Pos	Neg	Neg	2.5
BV0012	na	na	na	Pos	Pos	7
BV0047	na	na	na	Pos	Pos	7
BV0048	na	na	na	Pos	Pos	7

*Abbreviations*: *Pos* blood sample positive with *msp2* qPCR; *Neg* blood sample negative with *msp*2 qPCR; *na* not applicable (no sampling at this date)

#### German samples

The number of positive cattle at each sampling date varied from one (5%) to seven (35%) (Table 2). Altogether, 26/120 (21.7%) samples were found positive for specific *A. phagocytophilum* DNA with *msp2*-real-time PCR. One positive sample from the study performed in 2010 [16], and eight samples from five cows sampled in 2011 [17] were available for genotyping (Table 4).

**Table 4:** German samples used for molecular typing and dates of sampling

Bovine ID	Dates of positive samples used for typing		Time span between the two positive results
	First sampling date (year/month/day)	Second sampling date (year/month/day)	
BV34	2010/10/17	na	na
BV46	2011/05/22	2011/08/21	3 months
BV49	2011/05/19	na	na
BV57	2011/06/04	na	na
BV58	2011/05/17	2011/07/09	1.5 months
BV61	2011/06/22	2011/07/09	3 weeks

*Abbreviation*: *na* not applicable

### Genotyping and polymorphism analysis

Amplicons obtained with external or internal primers were sent for sequencing. For six loci (*typA*, *ctrA*, *msp4*, *pleD*, *recG*, and *polA*), the number of available sequences varied from 35 to 40 (all samples generated results for the *typA* locus). Sequence lengths after alignment ranged from 309 (*recG*) to 597 bp (*pleD*) (Table 5). Regarding *groEL*, *gyrA*, and *ankA*, only few samples gave sequencing results. Consequently, consecutive analyses were made using the loci *typA*, *ctrA*, *msp4*, *pleD*, *recG*, and *polA.*

**Table 5:** Genetic diversity observed at each locus. Amplicon lengths with external or internal primers were obtained with Primer-BLAST (NCBI), apart from the PCR products with *recG* external primers, and *polA* external and internal primers, which were estimated on agarose gels

	*typA*	*ctrA*	*msp*4	*pleD*	*recG*	*polA*
No. of complete sequences	40	39	36	36	35	37
No. of sequences without ambiguities	31	39	28	33	33	31
No. of sequences with ambiguities (%)	9 (22.5)	0 (0)	8 (22.2)	3 (8.3)	2 (5.7)	6 (16.2)
Amplicon length with external primers (bp)	1455	1453	558	1102	515	~490
Amplicon length with internal primers (bp)	550	574	471	na	~480	~460
Alignment length (bp)	385	317	337	597	309	331
No. of polymorphic sites	5	6	11	20	6	7
Proportion of polymorphic sites	1.30	1.89	3.26	3.35	1.94	2.11
No. of alleles at each locus	7	6	7	3	8	8

*Abbreviation*: *na* not applicable

Loci with the highest percentage of polymorphic sites were *pleD* (3.35%) and *msp4* (3.26%). The number of alleles for each locus varied from three (*pleD*) to eight (*polA* and *recG*). In 42% of French samples (13/31), double chromatograms peaks were encountered in up to four loci (*typA*, *msp4*, *pleD*, *recG*, and/or *polA*), which were confirmed by repeat sequencing on new amplicons. Ambiguities were only identified at particular polymorphic sites. In those samples with double peaks, the majority occurred at either *typA* or *msp4* loci, and five samples had ambiguous sites at both loci (Tables 5, 6). We excluded all samples with sequences presenting at least one ambiguous site from subsequent analyses, to avoid incongruence between loci.

**Table 6:** Complete sequencing results for the six loci and ambiguity occurrences. Those cattle for whom all six loci sequences were obtained are in bold. The strain clustering within the two major groups, based on the concatenation of five (all but *msp4*) or six loci, is indicated in the last column

Bovine ID	Sampling date (year/month/day)	Date of birth	*typA*	*ctrA*	*msp4*	*pleD*	*recG*	*polA*	Group in NJ tree
Twice-positive French samples									
**BV2802** **(Bull)**	**2014/10/09**	**2011/11/27**	**OK**	**OK**	**OK**	**OK**	**OK**	**OK**	**A**
**BV2802** **(Bull)**	**2016/03/04**		**OK**	**OK**	**OK**	**OK**	**OK**	**OK**	**B**
**BV9949**	**2016/03/04**	**2014/03/12**	**OK**	**OK**	**OK**	**OK**	**OK**	**OK**	**A**
BV9949	2016/05/17		1 AS	OK	OK	OK	OK	OK	
BV0001	2016/03/04	2015/04/05	1 AS	OK	6 AS	OK	OK	2 AS	
BV0001	2016/05/17		3 AS	OK	9 AS	19 AS	OK	1 AS	
BV0006	2016/03/04	2015/04/27	1 AS	OK	OK	OK	OK	OK	
BV0006	2016/05/17		OK	OK	9 AS	OK	OK	2 AS	
BV0016	2016/03/04	2015/11/14	OK	na	4 AS	OK	OK	na	
BV0016	2016/05/17		OK	OK	OK	OK	na	2 AS	
**BV0012**	**2016/10/25**	**2015/07/09**	**OK**	**OK**	**OK**	**OK**	**OK**	**OK**	**B**
**BV0012**	**2017/05/16**		**OK**	**OK**	**OK**	**OK**	**OK**	**OK**	**A**
**BV0047**	**2016/10/25**	**2016/08/29**	**OK**	**OK**	**OK**	**OK**	**OK**	**OK**	**A**
**BV0047**	**2017/05/16**		**OK**	**OK**	**OK**	**OK**	**OK**	**OK**	**A**
**BV0048**	**2016/10/25**	**2016/08/31**	**OK**	**OK**	**OK**	**OK**	**OK**	**OK**	**A**
**BV0048**	**2017/05/16**		**OK**	**OK**	**OK**	**OK**	**OK**	**OK**	**B**
Other French samples									
**BV1551**	**2014/10/09**	**2003/01/02**	**OK**	**OK**	**OK**	**OK**	**OK**	**OK**	**B**
**BV9932**	**2014/10/09**	**2013/05/03**	**OK**	**OK**	**OK**	**OK**	**OK**	**OK**	**A**
BV9940	2014/10/09	2013/05/16 (dead 2015/02/23)	2 AS	OK	3 AS	19 AS	na	OK	
BV9942	2014/10/09	2013/05/27	OK	OK	OK	OK	na	OK	
BV9952	2014/10/09	2014/04/13	3 AS	OK	5 AS	OK	na	OK	
BV9873	2014/10/09	2012/03/11 (dead 2016/06/09)	OK	OK	2 AS	na	na	na	
BV9959	2016/03/04	2014/05/05	OK	na	OK	na	OK	OK	
**BV2193**	**2016/05/17**	**na**	**OK**	**OK**	**OK**	**OK**	**OK**	**OK**	**A**
**BV2302**	**2016/05/17**	**na**	**OK**	**OK**	**OK**	**OK**	**OK**	**OK**	**B**
**BV3559**	**2016/05/17**	**na**	**OK**	**OK**	**OK**	**OK**	**OK**	**OK**	**A**
BV0013	2016/05/17	2015/07/09	1 AS	OK	10 AS	OK	2 AS	1 AS	
**BV9955**	**2016/05/17**	**2014/04/15**	**OK**	**OK**	**OK**	**OK**	**OK**	**OK**	**B**
BV0027	2017/05/16	2016/04/13	5 AS	OK	OK	OK	OK	OK	
BV0049	2017/05/16	2016/10/05	5 AS	OK	OK	19 AS	1 AS	2 AS	
BV0056	2017/05/16	2016/11/23	OK	na	OK	OK	OK	OK	
Twice-positive German samples									
BV46	2011/05/22	2008/08/27	OK	OK	na	OK	OK	OK	**A**
**BV46**	**2011/08/21**		**OK**	**OK**	**OK**	**OK**	**OK**	**OK**	**A**
BV58	2011/05/17	2008/08/18	OK	OK	na	OK	OK	OK	**A**
BV58	2011/07/09		OK	OK	OK	OK	OK	na	A
BV61	2011/06/22	2008/10/15	OK	OK	na	OK	OK	OK	**A**
BV61	2011/07/09		OK	OK	na	OK	OK	OK	**A**
Other German samples									
**BV49**	**2011/05/19**	**2008/03/12**	**OK**	**OK**	**OK**	**OK**	**OK**	**OK**	**A**
**BV34**	**2010/10/17**	**2007/10/14**	**OK**	**OK**	**OK**	**OK**	**OK**	**OK**	**A**
**BV57**	**2011/06/04**	**2007/11/27**	**OK**	**OK**	**OK**	**OK**	**OK**	**OK**	**A**

*Abbreviations*: *OK* complete sequence for the locus, without double peaks, *AS* ambiguous sites, *na* not available

Nineteen samples (four German samples from four cows, and 15 French samples from 11 cows) generated complete profiles for the six markers, without any ambiguities (Table 6).

The sequences obtained in this study are available in the GenBank database under the accession numbers MF580591–MF580636.

### Phylogenetic analyses

**Fig. 1:**
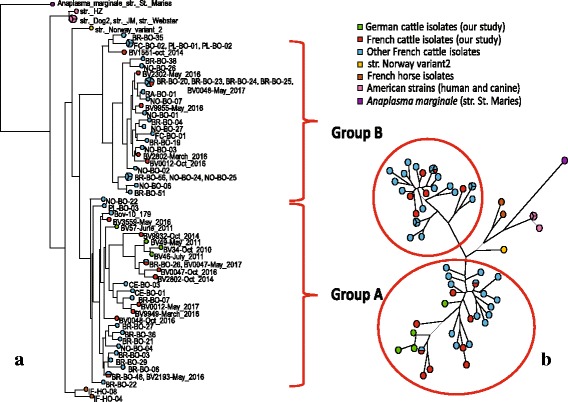
Neighbor-joining trees obtained with six loci concatenation. Each circle represents a unique sequence type. Logarithmic scale is used. To increase the readability of the second tree (**b**), names of samples have been removed, but can be found in the first tree (**a**). German cattle samples are in green, whereas the French are in red (our study), or in blue (Chastagner et al. study [9]). The two groups of cattle strains are identified and are clearly distinct from horse strains and American strains

Nineteen samples from our study and sequences from Chastagner et al. [7] were included in the phylogenetic tree based on the concatenation of six loci (Fig. 1). Building this tree allowed identifying groups of isolates and variants within groups. Variants are defined here as bacteria that evolved from the same bacterial cell. In the case of intra-cellular bacteria such as *A. phagocytophilum*, mutations (and possibly recombinations) occur at a high rate in single strains within an infected host. A group therefore gathers together variants that have highly similar profiles according to phylogenetic analyses, and that probably share the same ancestor. Two groups of bovine variants (A and B) were obtained, which were clearly distinct from the American strains, from the French horse strains, and from the Norwegian sheep strain (Table 6). Both groups were detected within the French herd, whereas only group A was present in the German herd. Interestingly, four French cows with positive results at two different time points enabled the comparison of two entire profiles from the same animal. Three of these cows (BV0012, BV0048 and BV2802), had a different profile at each time point which clustered into separate groups. Conversely, the two profiles from the fourth cow (BV0047) both clustered into group A.

Four German samples with indecipherable *msp4* sequences were added to the above-mentioned samples in order to build a phylogenetic tree based on concatenation of five loci (Additional file 2: Figure S1). Clustering reconfirmed the presence of two cattle groups, and the additional German samples clustered into group A. Interestingly, the profiles from BV46 and BV61 which were positive at two time points, differed between the two sampling dates. The two heifers were first infected by a variant from the same group, and subsequently (three weeks for BV61 and three months for BV46) they were both infected by another variant, which again shared the same profile.

The tree based on *typA*, *ctrA*, *msp4*, and *pleD* concatenation (Additional file 3: Figure S2) was consistent with the two other trees, whereas bovine groups became mixed when using the other loci concatenations (Additional file 4 Figure S3, Additional file 5: Figure S4, Additional file 6: Figure S5). Sequences from roe deer on one hand, and from dogs and horses on the other, also clustered into two distinct groups.

## Discussion

Our results reveal the co-existence of two major groups of bovine *A. phagocytophilum* strains among all cattle samples analyzed in this study. These groups did not associate with geographical origin, as cattle from the study by Chastagner et al. [9] originated from several French regions and clustered into either group. Moreover, these two groups remained stable over time according to MLST profiles, as identical sequences were obtained 31 months apart in the French herd. Although the bovine strains of these two groups are clearly distinct in NJ trees, the variant diversity within them remains high, as has already been described [20]. The variability of *A. phagocytophilum* strains within defined sheep flocks was already mentioned [10, 21–23]. Even so, there is no molecular typing data from any longitudinal study of *A. phagocytophilum* infection in cattle herds.

The six loci used in this study were sufficient to discriminate isolates belonging to the two groups, variants within the same group and successive isolates from the same animal. Indeed, the two strains from BV61, which were obtained only three weeks apart, were separated by using the concatenation of only five markers (*typA*, *ctrA*, *pleD*, *recG*, and *polA*). Given the irregularity in the collection of samples over time and given the possible interference of antibiotic treatments, this study was not intended to determine the intra-herd infection rate but instead to collect data on the molecular characteristics of circulating *A. phagocytophilum* strains within one given herd. Indeed, two groups, with variants within them, were clearly observed. Nevertheless, the use of additional markers could help to discriminate isolates obtained from the same animal at very close successive dates. Thus, another longitudinal study with more frequent and regular sampling would be very interesting in order to study in depth the succession of strains and/or the evolution of strains with time within one given animal.

The two groups of *A. phagocytophilum* strains co-circulated within the French farm, whereas only one was observed in the German herd. The presence of variants from only one group in the German herd could be related to the fact that they are dairy cows (red and black Holsteins, German Simmental and hybrids), whereas the French herd are beef cattle (Limousines). Dairy cows are more confined, have less extensive grazing pastures, and return every day to the stable, so may be less likely to contract infections by different *A. phagocytophilum* strains through tick bites*.*

In addition, in 42% of French samples (13/31), double chromatogram peaks were encountered. The presence of these ambiguities suggests the simultaneous occurrence of two *A. phagocytophilum* profiles resulting from two different alleles at some positions. In some cases, these double peaks could be the consequence of punctual mutations occurring during a prolonged infection by a single strain that evolved over time within the host, leading to a final co-existence of two variants. This is likely the case when only one or two ambiguities at a single locus are observed in a sample. Nevertheless, other cases were suggestive of simultaneous infection by two or more distinct strains, as several samples contained sequences with many successive ambiguities (5/5 for *typA*, 10/11 for *msp4*, and 19/20 for *pleD*). This could result from the simultaneous transmission of several strains by the same tick, or from several ticks biting the same cow, leading to several independent inoculations of different strains. Interestingly, four of the eight French cattle that were positive at two different time points presented ambiguities in up to four loci, and for three cows, this occurred on both sampling dates. In future studies, performing PCR products cloning could lead to the identification of strains that are associated in multiple infections and could help to investigate whether specific strains are more likely to be present in simultaneous infections.

The presence of multiple infections has already been described [9, 12, 21, 23, 24] and is considered to be a major drawback in typing methods [12]. The MLST study of Huhn et al. [12] demonstrated that double infections are more frequently found in wild ruminants, and another study indicated that 60.7% of infected French roe deer harbored two or three genetic variants [24]. Chastagner et al. [9] reported that approximately 30% of roe deer samples presented multiple infections, compared to very few cattle samples. In addition, in a longitudinal study involving lambs, only 4 out of 85 PCR-positive samples (4.7%) were infected with multiple *A. phagocytophilum* strains [21]*.* In our study, more than 40% of *A. phagocytophilum* sequences obtained from cattle in France presented double peaks, a higher frequency than that which was previously reported [9, 12]. Assuming that ticks can harbor several *A. phagocytophilum* strains, animals with frequent tick exposure could be at risk of multiple infections. In Sologne, ticks were frequently found on cattle, so it was assumed that infection pressure with multiple strains could be high. Conversely, none of the *A. phagocytophilum* sequences obtained from cattle in Germany presented double peaks. This could be due to the fact that all variants circulating within the farm belonged to the same group. Nevertheless, such typing methods are only able to detect simultaneous infections by multiple strains if one variant does not dominate the pool, so the number of multiple infections could be underestimated.

This work also raises the question whether cattle can act as reservoirs for *A. phagocytophilum*. Eight French cattle and three German cows remained positive after several weeks or months. Three French cattle (BV0012, BV0048, and BV2802) were found to be successively infected by strains from each group, but the order of infection was not always the same (group A strain then group B strain, or vice versa) (Table 6). These results demonstrate that neither of the two groups appears to be predominant, and instead may favor reinfection by a different variant, which strongly suggests the existence of complex interactions between cattle and *A. phagocytophilum*, as well as a lack of cross protection against other *A. phagocytophilum* strains. Conversely, for BV0047 (French heifer), BV46 and BV61 (German heifers), both the initial and subsequent infective strains belonged to the same group and were genetically similar. These results favor a long-term infection by the same variant, which could have evolved with time, perhaps to counteract the host immune response. It is noteworthy to mention here that one of the two German samples (BV61) also displayed another *msp2* sequence although samples were taken only three weeks apart [13]. Future work should aim to sample animals daily or weekly in order to determine if long-term infections result from the evolution and divergence of the initial strain and/or by reinfection with another variant.

Moreover, as several animals were infected at each sampling time point, it could be hypothesized that a reservoir could exist at the herd scale. Such a reservoir would facilitate tick reinfection following tick inactivity periods, subsequent transmission to other cattle and the circulation of various strains within the herd. An experimental study in sheep demonstrated that *A. phagocytophilum* can persist in the tissues of this theoretical reservoir, even though blood PCR analyses failed to detect infection [25]. It has also been shown that sheep are efficient reservoirs of *A. phagocytophilum* even during the post-acute phase of infection [26], and thus these bacteria could periodically circulate in the blood and infect feeding ticks. Further studies are required to confirm the hypothesis that cattle could be considered as reservoirs, whether via long-term *A. phagocytophilum* persistence at the animal scale, and/or via active *A. phagocytophilum* circulation at herd scale, taking into account strain evolution in infected animals. This hypothesis is already reinforced by both our own results and by several previous studies.

First, in our study, none of the French and German bovine strains clustered with *A. phagocytophilum* strains from other host species, which clustered into two distinct phylogenetic groups, separating horse and dog sequences from roe deer sequences. This was not unexpected as only a few cattle sequences had been associated with these two groups in a previous study [9]. Secondly, recent multiple-locus variable number tandem repeat analysis showed that cattle strains could be divided into two groups [10]. In the aforementioned study, the first group clustered only with red deer (*Cervus elaphus*) strains, whereas the second clustered with strains isolated from various host species (roe deer, horses, dogs, humans, and sheep)*.* Cattle may represent accidental hosts for variants belonging to the second group, which also included sheep, another domestic ruminant species already recognized as an *A. phagocytophilum* reservoir species for its own strains [8]. These data are very interesting as cattle and sheep do not usually share grazing pastures in France and Germany. It is therefore unlikely that sheep are potential reservoirs of cattle strains in these countries. Conversely, both red deer (suspected reservoir hosts of cattle variants [8–13]) and cattle herds themselves (current results) could be implicated according to ecological contexts, in the long term persistence of cattle-infecting strains. This role does not contradict the development of protective immunity at the scale of the individual animal [27], as indicated by the young mean age of positive cattle in our study. In recently infected animals, there could be a window of time where *A. phagocytophilum* may escape the immune response via antigenic variations and other mechanisms. Moreover, according to our results, it seems likely that a new strain is able to co-exist with another strain and/or with the immune response previously developed by a cow against another strain.

## Conclusions

For the first time, our study demonstrates the co-circulation of two major groups of *A. phagocytophilum* strains within a cattle herd in France and the presence of multiple variants within the group(s) circulating in France and in Germany. Eleven cows were found infected several weeks to several months after previous sampling, with a variant belonging or not to the same group. Moreover, 42% of French cattle presented ambiguous sequence reads at one or several loci, suggestive of simultaneous infections with multiple strains. Interestingly, at each sampling time point, *A. phagocytophilum* DNA was detected in blood samples from several cattle without clinical signs. This suggests that cattle could play the role of reservoir for strains with bovine host tropism, at least at the herd level.

## Additional files

Additional file 1: Table S1. DNA samples from Chastagner et al. [9] used for the alignments. The available sequences for each locus are indicated by an “X”. (XLSX 12 kb)

Additional file 2:Figure S1. NJ tree obtained using the concatenation of *typA*, *ctrA*, *pleD*, *recG*, and *polA*. Legends as in Fig. 1. (PDF 1383 kb)

Additional file 3: Figure S2. NJ tree obtained using the concatenation of *typA*, *ctrA*, *msp4*, and *pleD*. Legends as in Fig. 1. (PDF 1399 kb)

Additional file 4: Figure. S3. NJ tree obtained using the concatenation of *typA*, *ctrA*, *msp4*, *and recG.* Legends as in Fig. 1. (PDF 1395 kb)

Additional file 5: Figure S4. NJ tree obtained using the concatenation of *typA*, *ctrA*, *msp4*, and *polA.* Legends as in Fig. 1. (PDF 1434 kb)

Additional file 6: Figure S5. NJ tree obtained using the concatenation of *typA*, *ctrA*, and *msp4.* Legends as in Fig. 1. (PDF 1438 kb)

## Abbreviations

MLST: Multi-locus sequence typing
msp2: Major surface protein 2
NJ: Neighbor joining
TBF: Tick-borne fever
