# Supplementary material for: Co-circulation of different A. phagocytophilum variants within cattle herds and possible reservoir role for cattle
Source: Parasit Vectors. 2018 Mar 9;11:163. doi: 10.1186/s13071-018-2661-7 (PMC5845262; doi:10.1186/s13071-018-2661-7)
Supplement: Supplementary file 2 — NJ tree obtained using the concatenation of typA, ctrA, pleD, recG, and polA. Legends as in Fig. 1. (PDF 1383 kb) [file 13071_2018_2661_MOESM2_ESM.pdf]

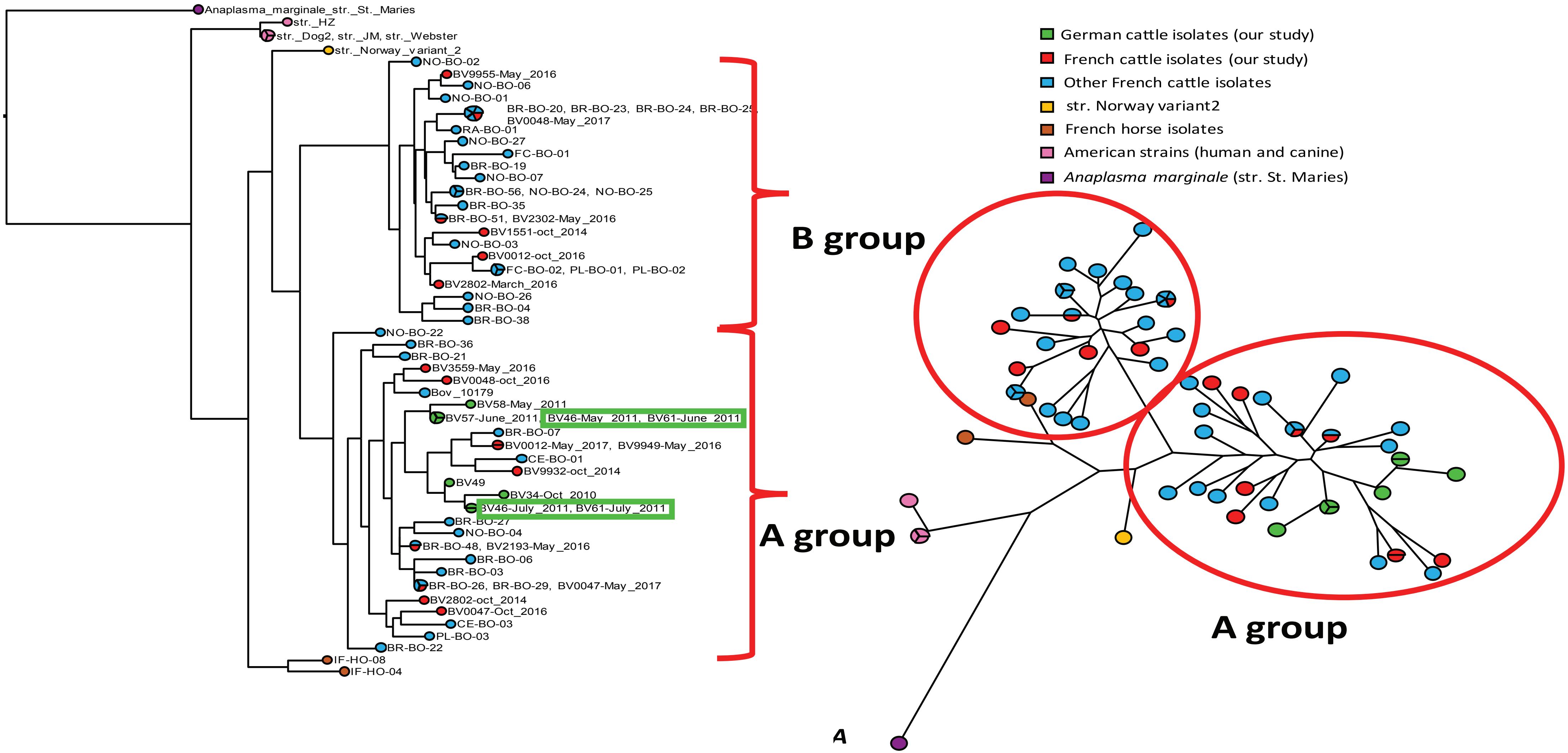

Anaplasma_marginale_str._St._Maries
str._HZ
str._Dog2, str._JM, str._Webster
str._Norway_variant_2
NO-BO-02
BV9955-May_2016
NO-BO-06
NO-BO-01
BR-BO-20, BR-BO-23, BR-BO-24, BR-BO-25,
BV0048-May_2017
RA-BO-01
NO-BO-27
FC-BO-01
BR-BO-19
NO-BO-07
BR-BO-56, NO-BO-24, NO-BO-25
BR-BO-35
BR-BO-51, BV2302-May_2016
BV1551-oct_2014
NO-BO-03
BV0012-oct_2016
FC-BO-02, PL-BO-01, PL-BO-02
BV2802-March_2016
NO-BO-26
BR-BO-04
BR-BO-38
NO-BO-22
BR-BO-36
BR-BO-21
BV3559-May_2016
BV0048-oct_2016
Bov_10179
BV58-May_2011
BV57-June_2011, BV46-May_2011, BV61-June_2011
BR-BO-07
BV0012-May_2017, BV9949-May_2016
CE-BO-01
BV9932-oct_2014
BV49
BV34-Oct_2010
BV46-July_2011, BV61-July_2011
BR-BO-27
NO-BO-04
BR-BO-48, BV2193-May_2016
BR-BO-06
BR-BO-03
BR-BO-26, BR-BO-29, BV0047-May_2017
BV2802-oct_2014
BV0047-Oct_2016
CE-BO-03
PL-BO-03
BR-BO-22
IF-HO-08
IF-HO-04
B group
A group
German cattle isolates (our study)
French cattle isolates (our study)
Other French cattle isolates
str. Norway variant2
French horse isolates
American strains (human and canine)
Anaplasma marginale (str. St. Maries)
A group
A
