# Supplementary figures and images for: Co-circulation of different A. phagocytophilum variants within cattle herds and possible reservoir role for cattle
Source: Parasit Vectors. 2018 Mar 9;11:163. doi: 10.1186/s13071-018-2661-7 (PMC5845262; doi:10.1186/s13071-018-2661-7)

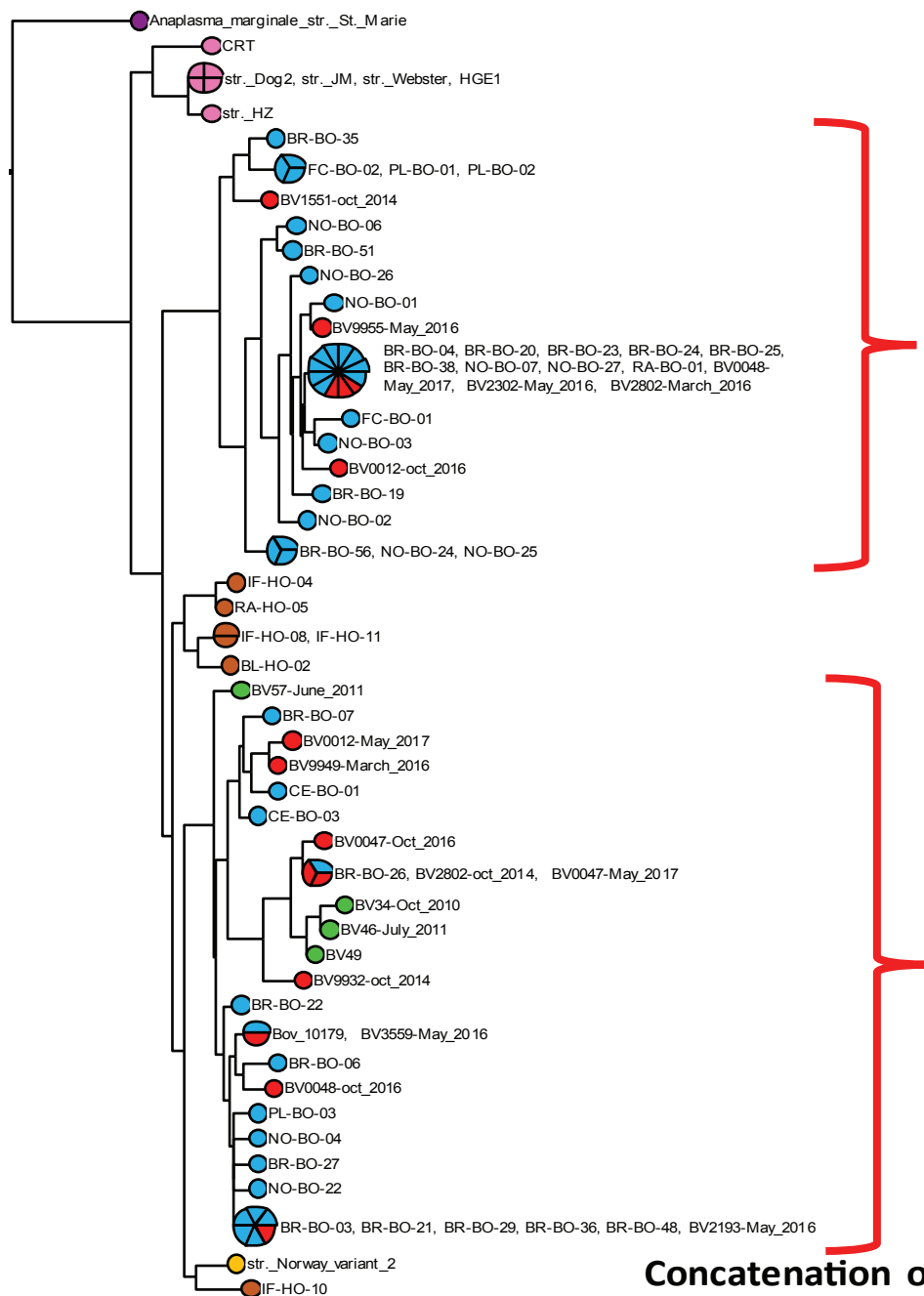

**B group**

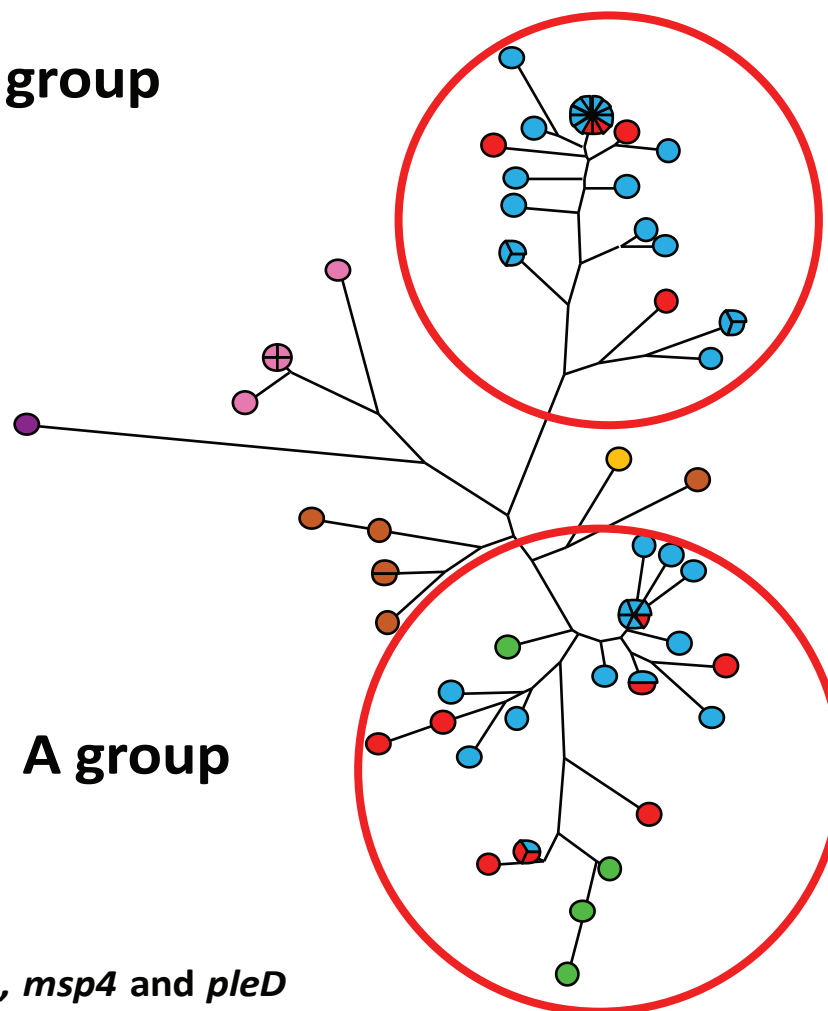

**A group**

Concatenation of *typA*, *ctrA*, *msp4* and *pleD*

Supplement: Supplementary file 3 — NJ tree obtained using the concatenation of typA, ctrA, msp4, and pleD. Legends as in Fig. 1. (PDF 1399 kb) [file 13071_2018_2661_MOESM3_ESM.pdf]
