# Supplementary material for: Co-circulation of different A. phagocytophilum variants within cattle herds and possible reservoir role for cattle
Source: Parasit Vectors. 2018 Mar 9;11:163. doi: 10.1186/s13071-018-2661-7 (PMC5845262; doi:10.1186/s13071-018-2661-7)
Supplement: Supplementary file 6 — NJ tree obtained using the concatenation of typA, ctrA, and msp4. Legends as in Fig. 1. (PDF 1438 kb) [file 13071_2018_2661_MOESM6_ESM.pdf]

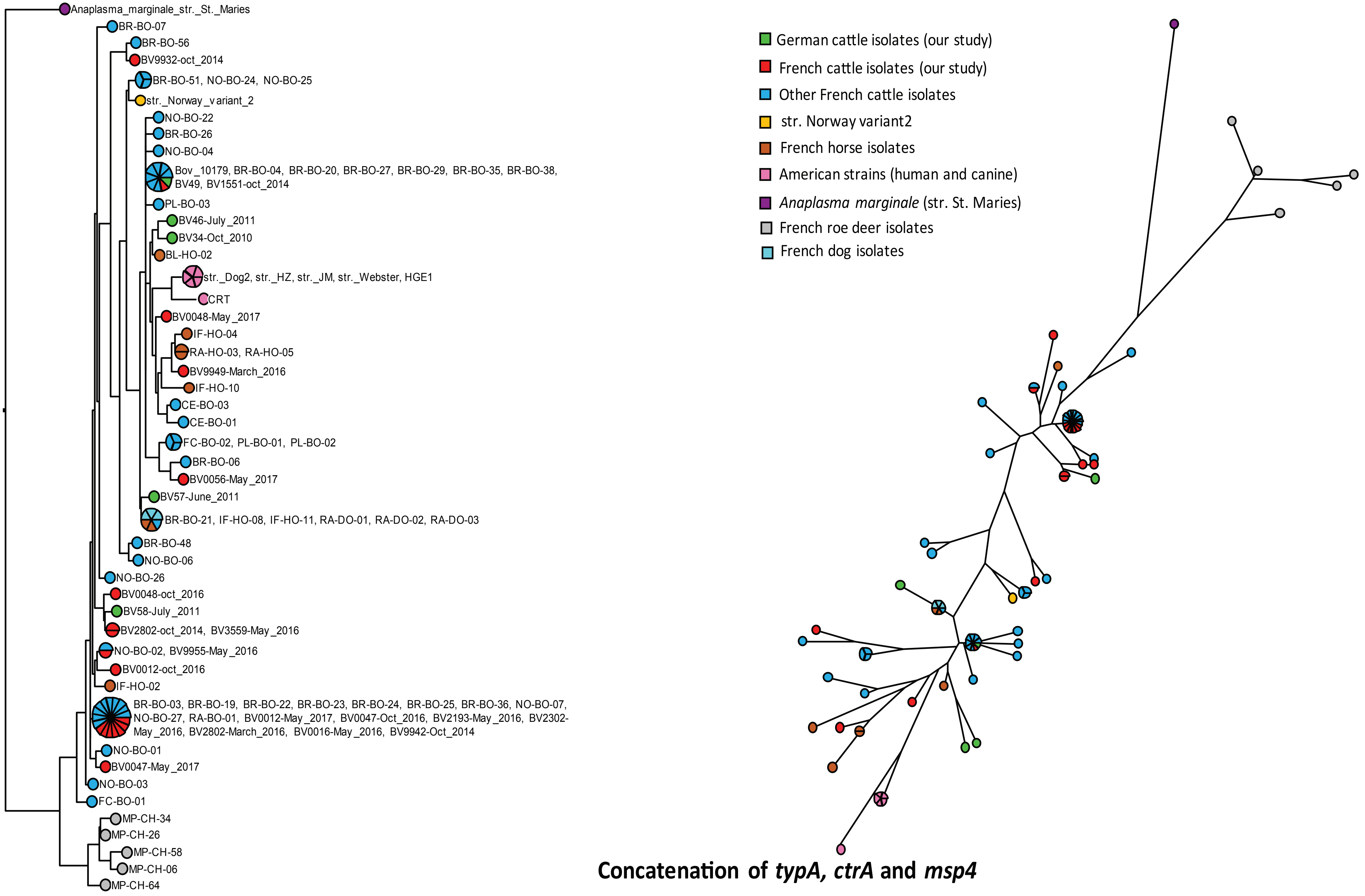
Anaplasma_marginale_str._St._Maries
BR-BO-07
BR-BO-56
BV9932-oct_2014
BR-BO-51, NO-BO-24, NO-BO-25
str._Norway_variant_2
NO-BO-22
BR-BO-26
NO-BO-04
Bov_10179, BR-BO-04, BR-BO-20, BR-BO-27, BR-BO-29, BR-BO-35, BR-BO-38, BV49, BV1551-oct_2014
PL-BO-03
BV46-July_2011
BV34-Oct_2010
BL-HO-02
str._Dog2, str._HZ, str._JM, str._Webster, HGE1
CRT
BV0048-May_2017
IF-HO-04
RA-HO-03, RA-HO-05
BV9949-March_2016
IF-HO-10
CE-BO-03
CE-BO-01
FC-BO-02, PL-BO-01, PL-BO-02
BR-BO-06
BV0056-May_2017
BV57-June_2011
BR-BO-21, IF-HO-08, IF-HO-11, RA-DO-01, RA-DO-02, RA-DO-03
BR-BO-48
NO-BO-06
NO-BO-26
BV0048-oct_2016
BV58-July_2011
BV2802-oct_2014, BV3559-May_2016
NO-BO-02, BV9955-May_2016
BV0012-oct_2016
IF-HO-02
BR-BO-03, BR-BO-19, BR-BO-22, BR-BO-23, BR-BO-24, BR-BO-25, BR-BO-36, NO-BO-07, NO-BO-27, RA-BO-01, BV0012-May_2017, BV0047-Oct_2016, BV2193-May_2016, BV2302-May_2016, BV2802-March_2016, BV0016-May_2016, BV9942-Oct_2014
NO-BO-01
BV0047-May_2017
NO-BO-03
FC-BO-01
MP-CH-34
MP-CH-26
MP-CH-58
MP-CH-06
MP-CH-64
German cattle isolates (our study)
French cattle isolates (our study)
Other French cattle isolates
str. Norway variant2
French horse isolates
American strains (human and canine)
Anaplasma marginale (str. St. Maries)
French roe deer isolates
French dog isolates
Concatenation of *typA, ctrA* and *msp4*
